# Determination of HER2 status using both serum HER2 levels and circulating tumor cells in patients with recurrent breast cancer whose primary tumor was HER2 negative or of unknown HER2 status

**DOI:** 10.1186/bcr1783

**Published:** 2007-10-26

**Authors:** Tanja Fehm, Sven Becker, Silke Duerr-Stoerzer, Karl Sotlar, Volkmar Mueller, Diethelm Wallwiener, Nancy Lane, Erich Solomayer, Jonathan Uhr

**Affiliations:** 1 Department of Obstetrics and Gynecology, University of Tuebingen, Calwerstrasse, D-72076 Tuebingen, Germany; 2 Department of Pathology, University of Tuebingen, Liebermeisterstrasse, D-72076 Tuebingen, Germany; 3 Department of Gynecology, University Medical Center Hamburg-Eppendorf, Martinistrasse, 20246 Hamburg, Germany; 4 Cancer Immmunobiology Center, University of Texas Southwestern Medical School, Harry Hines Boulevard, Dallas, Texas 75390, USA

## Abstract

**Introduction:**

At the time when metastatic disease is identified, assessment of human epidermal growth factor receptor (HER)2 status might help to optimize treatment decisions if HER2 status was not determined at first diagnosis and if HER2 positivity has been acquired during disease progression. Within this context, determination of serum HER2 or evaluation of HER2 status in circulating tumor cells (CTCs) may be of clinical relevance because metastatic tissue may be difficult to obtain for analysis as a result of its localization. The aim of this study was therefore to determine the HER2 status in serum and corresponding CTCs in patients with metastatic breast cancer whose primary tumors were HER2 negative or of unknown HER2 status.

**Methods:**

Blood samples were obtained from 77 metastatic breast cancer patients with negative (*n *= 44) or unknown (*n *= 33) HER2 status. Serum HER2 was determined using a commercial HER2/neu ELISA kit. CTCs were detected by slide-based assay using immunomagnetic enrichment and characterized by phenotyping and genotyping. Alternatively, a commercial kit, based on RT-PCR, was used to detect and characterize CTCs.

**Results:**

Twenty out of 77 patients with metastatic disease had elevated serum levels of HER2. Blood samples could be analyzed for the presence of CTCs in 67 patients. Eight out of 21 patients with detectable CTCs exhibited HER2 amplification. Twenty-three out of 77 patients were HER2 positive using at least one method. Concordance between HER2 status of CTCs and serum HER2 was observed in 15 of 21 patients (71%). In six patients conflicting results were obtained. Three patients with elevated serum HER2 status had HER2-negative CTCs, whereas three patients with HER2-positive CTCs had normal serum HER2 levels.

**Conclusion:**

A subgroup of patients with initially negative or unknown HER2 status can have elevated serum HER2 levels and/or HER2-positive CTCs at the time of development of metastatic disease. Although only a small number of patients were studied, our observations are of clinical relevance because, currently, these patients do not have access to HER2-targeted therapy.

## Introduction

Human epidermal growth factor receptor (HER)2 is a transmembrane tyrosine kinase growth receptor protein that is encoded by a proto-oncogene located on chromosome 17q21. The HER2 proto-oncogene is amplified or over-expressed in approximately 20% to 25% of invasive primary breast cancers [1-3]. Positive HER2 status has been linked with aggressive tumor behaviour and resistance to cytotoxic and endocrine therapies [3-5]. Patients with HER2 amplification or over-expression are eligible for treatment with trastuzumab (Herceptin^®^), a monoclonal antibody directed against HER2. Trastuzumab inhibits neoplastic cell proliferation *in vivo *and *in vitro *and enhances chemosensitivity [6,7]. It has been approved for clinical use in metastatic breast cancer. Based on recently reported results from four large trastuzumab trials (Herceptin Adjuvant trial, the North Central Cancer Treatment Group trial N9831, National Surgical Adjuvant Project-31, and Breast Cancer International Research Group 006), trastuzumab is also indicated in adjuvant therapy in HER2-positive primary breast cancer [8-10]. In addition to trastuzumab, other therapeutic strategies have recently been developed to target the HER2 protein, such as the tyrosine kinase inhibitor lapatinib, which appears to have clinical activity after failure of trastuzumab therapy [11].

The methods used to select patients for HER2-targeted therapy are based on immunohistochemical detection of HER2 over-expression and demonstration of HER2 gene amplification by fluorescence *in situ *hybridization (FISH) in primary tissue [12]. However, the antigenic profile of primary tumors may be different from that in metastatic disease. A discrepancy between the primary tumor and distant metastases was observed in 7% to 20% of cases [13-17]. Therefore, the reassessment of HER2 status at the time of disease progression might help to optimize treatment by identifying additional patients who could profit from trastuzumab or other therapeutic approaches targeted against HER2. Biopsy of the metastatic lesion may not be an option because of its location.

An alternative to invasive tissue analysis is to determine the serum HER2 status. The extracellular domain of the HER2 receptor is released after cleavage by ADAM (a disintegrin and metalloproteinase) metalloproteinases and can be determined in serum using ELISA [18]. Elevated serum HER2 levels are highly correlated with HER2 over-expression and amplification in tumor tissue [19]. Another possibility is the evaluation of HER2 status by immunohistochemistry or FISH of circulating tumor cells (CTCs), which are frequently detected in the blood of metastatic breast cancer patients (cell-based assay) [20]. These methods could also be helpful for patients with recurrent breast cancer of unknown HER2 status.

Determination of the HER2 status at primary diagnosis was not a standard procedure until the introduction of trastuzumab into routine clinical management of breast cancer patients in the 1990s in many centers, or even until data indicating an effect of adjuvant trastuzumab treatment emerged in 2005. Therefore, there remains a significant group of newly diagnosed metastatic patients with unknown HER2 status. Because storage of archival material is limited (for example, 10 years in Germany), primary tumor tissue will not be available for HER2 assessment in many cases. Therefore, the aim of the study was to identify metastatic patients with initially negative or unknown HER2 status who might benefit from HER2-targeted therapy because of HER2 positivity at the time of metastatic disease. Our approach was to determine the HER2 status in serum and in corresponding CTCs

## Materials and methods

### Patients

Seventy-seven breast cancer patients who were diagnosed with metastatic disease either at the Department of Gynecology/Obstetrics, Tuebingen (Germany) or at the Department of Cancer Immunobiology, University of Texas Southwestern Medical School (Dallas, TX, USA) were included in the analysis. All patients gave informed consent. The blood samples were obtained before initiation of or change in therapy. The study protocol was approved by the local ethics committee (A455/2004).

Inclusion criteria were as follows: breast cancer patients with first diagnosis of metastatic disease or disease progression; and either unknown (*n *= 33) or negative HER2 status (*n *= 44) of the primary tumor. In patients with unknown HER2 status (*n *= 33), primary archival tumor tissue was requested by the corresponding Department of Pathology for HER2 assessment.

### Evaluation of serum HER2

Blood was drawn into serum tubes and centrifuged at 1,000 *g *for 10 minutes at room temperature. Serum samples were stored at -70°C. Serum HER-2 levels were measured using the Oncogene Science HER-2 Microtiter ELISA (Oncogene Science, Cambridge, MA, USA), in accordance with the manufacturer's instructions. A cut-off of 15 ng/ml was chosen based on the findings of a previous study using the same assay [21]. The intra-assay coefficent of variation was less than 5% and the interassay coefficent of variation was 10%. All serum samples were analyzed in the laboratory of the Department of Gynecology/Obstetrics, Tuebingen, Germany.

### Evaluation of HER2 status of circulating tumor cells

Blood samples from the Department of Cancer Immunobiology (Dallas, TX, USA) were analyzed using the ferrofluid assay (method A). The CTC analysis in patients of the Department of Gynecology/Obstetrics (Tuebingen, Germany) was conducted using the AdnaTest BreastCancer test (AdnaGen AG, Langenhagen, Germany; method B). In 10 patients CTC analysis was not performed because the patients only agreed to serum HER2 analysis or the blood samples were not provided by the physicians in accordance with the standard operating procedures for tumor cell analysis (with respect, for example, to storage temperature and use of appropriate blood tubes).

#### Slide-based assay

CTCs were isolated and enriched using a slide-based assay using ferrofluid linked to antibody to EpCAM (epithelial adhesion molecule) and identified by phenotyping using antibodies against a pan-cytokeratin (C11; Sigma, Natick, MA, USA) and HER2 (Her 81; provided by Dr J Uhr, University of Texas Southwestern Medical School, Dallas, TX, USA). CTCs with HER2 over-expression were hybridized using a centromeric probe for chromosome 17 and a locus-specific probe for HER2 (Vysis, Downers Grove, IL, USA). Details of the CTC-based assay and the subsequent phenotyping and genotyping are described in detail elsewhere [20].

#### RT-PCR based assay

Alternatively, a commercial kit (AdnaTest BreastCancerSelect/Detect; AdnaGen AG) based on RT-PCR was used for CTC detection and assessment of HER2 status, in accordance with the manufacturer's instructions [22]. AdnaTest Breast cancerSelect/Detect detects CTCs with a specificity in excess of 90% and sensitivity greater than 50% above a cut-off of 0.15 ng/μl for any of the amplicons (epithelial adhesion molecule: GA733-2, tumor associated mucin: MUC-1, and HER2RT-PCR and FISH should yield comparable results because HER2 mRNA over-expression corresponds to gene amplification and HER2 over-expression, as has been demonstrated by Walch and coworkers [23].

### HER2 status of the metastatic tissue

Re-evaluation of HER2 status of metastatic tissue was not part of the study and was only performed when requested by the clinician. Immunohistochemical staining was performed on 3 to 5 μm thick sections of metastatic tissue using a commercial available ABC kit (Vectastain; Vector Laboratories, Burlingame, CA, USA). Primary antibodies (HER2, clone A0485; Dako, Glostrup, Denmark) were diluted in Tris-HCl (pH 7.5) and applied in accordance with the manufacturer's instructions. The degree of staining was scored by a certified pathologist in accordance with the method recommended for the DakoHercepTest™. If the Hercep score was 2+, then a FISH analysis was performed to determine HER2 gene amplification.

### Statistical analysis

The relationship between categorical variables and clinicopathologic factors was analyzed using the χ^2 ^test. Statistical analysis was performed by SPSS (version 11.5; SPSS Inc., Chicago, IL, USA). *P *values less than 0.05 were considered statistically significant.

## Results

### Clinical data of the metastatic breast cancer patients

Seventy-seven metastatic breast cancer patients were included in the analysis. Twenty-five patients had visceral metastases and 13 patients had bone metastases, whereas multiple (visceral and bone) metastases were present in 39 patients. The clinical data are summarized in Table 1. Forty-four patients had a HER2-negative primary tumor. In 33 patients the HER2 status of the primary tumor had not been determined initially. In patients with unknown HER2 status (*n *= 33), primary archival tumor tissue was requested from the corresponding Department of Pathology for HER2 assessment. Because the interval from first diagnosis exceeded 10 years in most of the breast cancer patients with unknown HER2 status, tissue was available for HER2 analysis from only 10 of these patients. In four cases archival tissue from a local recurrence was analyzed for HER2 status. In five of the 14 (36%) patients for whom HER2 status was missing, the primary tumor was found to be HER2 positive.

**Table 1:** Clinical data of patients

	Number	%
Age		
<50 years	17	22
≥50 years	60	78
Primary tumor size		
pT1	25	32
pT2-4	40	52
PTx	12	16
Nodal status		
N_o_	22	29
N_1_	39	51
Nx	16	21
Grading		
I to II	35	45
III	24	31
Unknown	18	23
Estrogen receptor status		
Negative	20	26
Positive	45	58
Unknown	12	16
Progesterone receptor status		
Negative	25	33
Positive	39	50
Unknown	13	17
HER2 status^a^		
Negative	44	57
Unknown	33	43
Localization of metastases		
Visceral	25	33
Bone	13	17
Multiple/other	39	51

A total of 77 patients were included in this study. ^a^Human epidermal growth factor receptor (HER)2 status at the time of study inclusion; in 14 patients HER2 status could be determined from archival tissue (five HER2-positive patients and nine HER2-negative patients).

### Serum HER2 levels

Twenty out of the 77 (26%) patients with metastatic disease had serum HER2 levels above 15 ng/ml. Twelve of these patients (45%) were initially HER2 negative. HER2 status was unknown in four of the 20 patients (20%). Elevated HER2 levels were not correlated with initial tumor size, estrogen receptor or progesterone receptor status, and localization of metastases (data not shown). The percentage of HER2-positive patients was the same in the group with unknown HER2 status as compared with the group with negative HER2 status (21% versus 23%). Only one of the five patients with HER2 positive tumors at the time of first diagnosis had a serum HER2 level below the cut-off. Data are summarized in Table 2.

**Table 2:** HER2 re-evaluation by serum HER2, CTC, and metastatic tissue

		Primary tissue		
		
HER2 evaluation	Total	HER2 negative	HER2 unknown	HER2 positive
Serum HER2 (*n *= 77)	77	53	19	5
HER2 negative	57	41 (77)	15 (79)	1 (20)
HER2 positive	20	12 (23)	4 (21)	4 (80)
CTC HER2 (*n *= 21)^a^	21	12	6	3
HER2 negative	13	8 (*6*7)	3 (50)	2 (67)
HER2 positive	8	4 (33)	3 (50)	1 (33)
Metastatic tissue (*n *= 10)^b^	10	8	2	-
HER2 negative	8	6 (75)	2 (100)	-
HER2 positive	2	2 (25)	0	-

^1^Fifty-six patients were circulating tumor cell (CTC) negative; in 10 patients no CTC evaluation could be performed. ^b^Metastatic tissue was only available in 10 patients. HER, human epidermal growth factor receptor.

### HER2 status of circulating tumor cells

Blood samples from 67 patients could be analyzed for presence of CTC. Fifty-one blood samples were analyzed using the RT-PCR based assay. In 16 cases immunomagnetic separation followed by cytocentrifugation was performed (slide-based assay). CTCs were identified in 21 out of 67 (31%) patients. The CTC detection rate did not correlate with the detection method (cell-based assay versus RT-PCR based assay). HER2 status of CTCs was assessed using either RT-PCR (*n *= 51) or double immunofluorescence followed by FISH (*n *= 16). HER2 amplification was present in eight out of 21 cases with CTCs (38%). Two of these patients were initially HER2 negative. In six of these patients the HER2 status of the primary tumor was unknown.

### Correlation between serum HER2 and HER2 status of circulating tumor cells

Twenty-three out of 77 (30%) patients had positive HER2 status based on either the HER2 status of CTCs or serum. Concordance between HER2 status of CTCs and serum HER2 was observed in 15 out of 21 patients (71%). In six patients conflicting results were obtained. Three patients with elevated serum HER2 status had HER2-negative CTCs, whereas three patients with HER2-amplified CTCs had normal serum HER2 levels (Table 3).

**Table 3:** Concordance between serum HER2 status and HER2 status of CTCs

	CTC HER2 negative	CTC HER2 positive	Total
Serum HER2 positive	3	5	8
Serum HER2 negative	10	3	13
Total	13	8	21^a^

^a^Concordance is 71%. CTC, circulating tumor cell; HER, human epidermal growth factor receptor.

### Determination of HER2 status by serum and metastatic tissue

Table 4 summarizes the results. HER2 status could be reassessed with metastatic tissue in 10 patients. Two patients switched from HER2-negative primary tumors to HER2-positive metastatic sites based on tissue-based immunologic analysis. Only one of these two patients had elevated serum levels and HER2-positive CTCs. In contrast, one patient with positive HER2 status by serum and cell-based assay was HER2 negative by tissue-based analysis.

**Table 4:** HER2 status by serum, CTC, and tissue

Patient ID	Serum HER2	HER2 CTC	HER2 tissue
#25	Negative	ND	Negative
#93	Negative	ND	Negative
#94	Negative	ND	Negative
#21	Negative	ND	Negative
#92	Negative	ND	Negative
#29	Negative	ND	Negative
#30	Negative	ND	Negative
#91	Positive	Positive	Negative
#12	Negative	Negative	Positive
#17	Positive	Positive	Positive

CTC, circulating tumor cell; HER, human epidermal growth factor receptor; ND, not detected.

## Discussion

Development of metastatic disease is a highly selective process. Only a small portion of tumor cells of the primary tumor have the ability to initiate metastatic growth at a different organ site. Additional genetic changes accompany tumor progression and may occur before the recurrence is detected. Therefore, the phenotype of metastatic disease may be quite different from that of the primary tumor. Thus, it is important to establish the HER2 status of the recurrent tumor. However, biopsies from the metastatic site for re-evaluation of phenotype are not routinely obtained. Moreover, the phenotype may vary between different sites of metastatic disease. For these reasons, noninvasive re-evaluation of phenotype by CTCs or shed soluble proteins are attractive alternatives and might reflect the 'phenotype of the total tumor load' more accurately. In addition, both serum HER2 and CTC testing has the additional potential of monitoring the effect of therapy.

Assessment of HER2 status using these methods is also important for patients with recurrent breast cancer who have unknown HER2 status. As explained above, there is a significant group of newly diagnosed patients with metastatic disease whose HER2 status is unknown because of lack of archival primary tumor tissue.

Therefore, in this prospective study the HER2 status was reassessed at the time of identification of metastatic disease by evaluating serum HER2 in 77 metastatic breast cancer patients with initially HER2 negative or unknown HER2 status. Blood samples in 67 patients could be analyzed for HER2-positive CTCs. In 10 cases biopsies could be obtained from the metastatic site for HER2 re-evaluation. To our knowledge, this is the first study that compares reassessment of HER2 status using such different approaches.

### Reassessment of HER2 status by HER2 shed antigen

The extracellular domain of the HER2 receptor is shed by proteolysis and can be detected in the serum of breast cancer patients. The most commonly used cut-off level is 15 ng/ml for the US Food and Drug Administration approved ELISA. Normal control individuals are serum HER2 negative based on this cut-off [20]. At the time of first diagnosis of metastatic disease, approximately 20% to 50% of patients have positive serum HER2 status [24,25]. Serum HER2 levels are correlated with HER2 tissue expression, suggesting that the frequency of HER2-positive metastases is higher than expected based on the reported frequency of HER2 positivity in primary tumors ranging from 15% to 30% [16,23].

Positive serum HER2 status at the time of metastatic disease has been linked to poor prognosis [26-28]. Moreover, several studies have indicated that positive serum HER2 status is predictive of therapy response to Herceptin [29-31]. Moreover, changes in serum HER2 levels correlate with clinical response to HER2-targeted therapy, as demonstrated by a recent pooled analysis of seven trials of first-line trastuzumab therapy [32]. Increasing levels indicate disease progression, whereas decreasing levels are associated with a therapeutic response or stable disease [32,33]. Currently, the serum HER2 test only has Food and Drug Administration approval for monitoring changes in serum HER2 levels in patients with metastatic breast cancer.

In our study, serum HER2 was determined in order to re-evaluate HER2 status in 77 metastatic breast cancer patients with HER2-negative (*n *= 53), HER2-positive (*n *= 5), and unknown HER2 status (*n *= 19) of the primary tumor. Of these patients, 21% were serum HER2 positive at the time of metastatic disease. Slightly higher positivity rates in initially HER2-negative breast cancer patients at the time of metastatic disease were observed by Anderson and coworkers (34%) [34] and Fehm and colleagues (34%) [35] using the serum test. Currently, such patients are not considered eligible for HER2-targeted therapy, but based on recent studies it is high likely that such individuals would respond to Herceptin [29].

### The clinical significance of HER2 positive circulating tumor cells

Tumors, including metastatic lesions, shed large numbers of tumor cells into the blood circulation [36], and the presence of CTCs is of biologic relevance in the metastatic setting. Based on the hypothesis that the phenotype of CTCs may reflect the phenotype of metastatic disease, characterization of CTCs may be useful for reassessment of HER2 status and additional therapeutic markers [20]. However, this option is limited to those patients with detectable CTCs. Reported positivity rates for CTCs in metastatic breast cancer patients range from 20% to 60% [37,38].

In our prospective study, in which 67 patients could be included, 21 patients had detectable CTCs (31%). HER2 was over-expressed in eight of these 21 patients (38%). Meng and coworkers [20] reassessed the HER2 status in 31 metastatic patients with CTCs. Nine out of 24 patients with initially HER2-negative tumors had HER2-positive cells; this rate (38%) is lower than our observed positivity rate. Four of these nine patients were treated with a Herceptin-containing chemotherapy regimen. Two of these patients exhibited partial or complete remission [20].

Hayes and colleagues [39] evaluated the number of HER2-positive CTCs during the course of treatment by flow cytometry in 19 metastatic breast cancer patients. During disease progression, the HER2 status of CTCs changed from negative to positive in all cases (*n *= 3). In contrast, patients responding to trastuzumab treatment lost their HER2-positive cell fraction. Meng and coworkers [40] analyzed the urokinase-type plasminogen activator receptor and HER2 gene status in individual breast cancer cells from metastatic breast cancer patients. Five out of 52 formerly HER2-negative cases acquired HER2 positivity. Interestingly, in two initially HER2-positive patients with disease progression, CTCs were negative for HER2 after treatment with trastuzumab.

The major caveat of all of these observational studies, including the present study, is the low number of patients included. Therefore, large clinical trials must be initiated to evaluate further whether acquisition of HER2-positive CTCs are predictive of clinical response to HER2-targeted therapy in a subgroup of patients with metastatic breast cancer.

### Correlation between serum HER2 and HER2-positive circulating tumor cells

Elevated serum HER2 levels are associated with HER2 over-expression and amplification in breast cancer tissue. Nevertheless, discordant results between serum and tissue can be obtained in a small subset of patients. Differences in serum HER2 concentrations for a given level of tissue HER2 expression may be caused by different levels of activators that are involved in HER2 cleavage, such as metalloproteinase [41]. Molina and colleagues [42] demonstrated that cleavage of the extracellular domain leads to increased phosphorylation of the intracellular tyrosine kinase, resulting in an increased receptor activation. However, both serum HER2 and HER2 over-expression are predictive of response to Herceptin therapy. The optimal method for predicting response to therapy is yet to be determined. In the present study we compared the HER2 status of CTCs and shed HER2. A similar rate of nonconcordance was reported in studies comparing serum and tissue HER2 levels in primary breast cancer [34,35]. These results support the hypothesis that HER2 shedding may reflect activity of the HER2 shedding machinery and therefore is not necessarily entirely associated with HER2 amplication and/or over-expression in tissue or CTCs. In a small subgroup of patients included in the present study, metastatic tissue could be tested for HER2 over-expression. In eight patients, serum HER2 and tissue-based HER2 analysis were negative. Two out of 10 (20%) patients had HER2-positive metastases. Only one of the two patients was HER2 positive by serum analysis and cell-based assay. However, relatively little attention has been paid to the observed heterogeneity of HER2 status within individual tumors, which may contribute to discordancy among different methods of measurement of HER2 status as well as to their correlation with conventional pathologic analysis.

## Conclusion

Our study indicates that in patients with initially negative or unknown HER2 status, elevated serum HER2 levels and/or HER2-positive CTCs can be detected at the time of identification of metastatic disease. However, nonconcordant results were obtained in 28% of patients using serum and CTCs for HER2 reassessment. Similar nonconcordance for HER2 levels occurs in primary breast carcinomas but may not occur with HER2 status of CTCs Therefore, we hypothesize that different mechanisms may account for shedding HER2 as opposed to its expression. Correlations between clinical responses to HER2-targeted therapy and findings with each method should be further studied to help determine when such treatment should be given.

## Abbreviations

CTC = circulating tumor cell; ELISA = enzyme-linked immunosorbent assay; FISH = fluorescence *in situ *hybridization; HER = human epidermal growth factor receptor; RT-PCR = reverse transcription polymerase chain reaction.

## Competing interests

The authors declare that they have no competing interests.

## Authors' contributions

TF, NL, JU, SDS, KS and ES made substantial contributions to conception and design of the study, or acquisition of data, or analysis and interpretation of data. TF, SB, DW, JU and VM were involved in drafting the manuscript or revising it critically for important intellectual content. All authors read and approved the final manuscript.

## References

[B1] Coussens L, Yang-Feng TL, Lioa YC, Chen E, Gray A, McGrath J, Seeburg PH, Libermann TA, Schlessinger J, Francke U (1985). Tyrosine kinase receptor with extensive homology to EGF receptor shares chromosomal location with neu oncogene. Science.

[B2] Schecter AL, Stern DF, Vaidyanathan L, Decker SJ, Drebin JA, Greene MI, Weinberg AR (1984). The neu oncogene : An erbB-related gene encoding a 185,000-M_r _tumor antigen. Nature.

[B3] Slamon DJ, Goldolphin W, Jones LA, Holt JA, Wong SG, Keith DE, Levin WJ, Stuart SG, Udove J, Ullrich A (1989). Studies of the HER-2/proto-oncogene in human breast cancer and ovarian cancer. Science.

[B4] Konecny G, Pauletti G, Pegram M, Untch M, Dandekar S, Aguilar Z, Wilson C, Rong HM, Bauerfeind I, Felber M (2003). Quantitative association between HER-2/neu and steroid hormone receptors in hormone receptor-positive primary breast cancer. J Natl Cancer Inst.

[B5] Moliterni A, Menard S, Valagussa P, Biganzoli E, Boracchi P, Balsari A, Casalini P, Tomasic G, Marubini E, Pilotti S, Bonadonna G (2003). HER2 overexpression and doxorubicin in adjuvant chemotherapy for resectable breast cancer. J Clin Oncol.

[B6] Pegram M, Hsu S, Lewis G, Pietras R, Beryt M, Sliwkowski M, Coombs D, Baly D, Kabbinavar F, Slamon D (1999). Inhibitory effects of combinations of HER-2/neu antibody and chemotherapeutic agents used for treatment of human breast cancers. Oncogene.

[B7] Muss HB, Thor AD, Berry DA, Kute T, Liu ET, Koerner F, Cirrincione CT, Budman DR, Wood WC, Barcos M (1994). c-erbB-2 expression and response to adjuvant therapy in women with node-positive early breast cancer. N Engl J Med.

[B8] Romond EH, Perez EA, Bryant J, Suman VJ, Geyer CE, Davidson NE, Tan-Chiu E, Martino S, Paik S, Kaufman PA (2005). Trastuzumab plus adjuvant chemotherapy for operable HER2-positive breast cancer. N Engl J Med.

[B9] Piccart-Gebhart MJ, Procter M, Leyland-Jones B, Goldhirsch A, Untch M, Smith I, Gianni L, Baselga J, Bell R, Jackisch C, Herceptin Adjuvant (HERA) Trial Study Team (2005). Trastuzumab after adjuvant chemotherapy in HER-2 positive breast cancer. N Engl J Med.

[B10] Viani GA, Afonso SL, Stefano EJ, De Fendi LI, Soares FV (2007). Adjuvant trastuzumab in the treatment of HER-2-positive early breast cancer: a meta-analyses of published randomized trials. BMC Cancer.

[B11] Johnston SRD, Leory A (2006). Lapatinib: a novel EGFR/HER2 tryosine kinase inhibitor for cancer. Drugs Today.

[B12] Wolff AC, Hammond ME, Schwartz JN, Hagerty KL, Allred DC, Cote RJ, Dowsett M, Fitzgibbons PL, Hanna WM, Langer A (2006). American Society of Clinical Oncology/College of American Pathologists Guideline Recommendations for Human Epidermal Growth Factor Receptor 2 Testing in Breast Cancer. J Clin Oncol.

[B13] Zidan J, Dashkovsky I, Stayerman C, Basher W, Cozacov C, Hadary A (2005). Comparison of HER2 overexpression in primary breast cancer and metastatic sites and its effect on biological targeting therapy of metastatic disease. Br J Cancer.

[B14] Edgerton S, Moore DH, Merkel D, Thor AD (2003). HER-2/neu/erbB-b2 status by immunohistochemistry and FISH: clonality and regression with recurrence and metastases. Appl Immunohistochem Mol Morphol.

[B15] Gancberg D, DiLeo A, Cardoso F, Rouas G, Pedrocchi M, Paesmans M, Verhest A, Bernard-Marty C, Piccart MJ, Larsimont D (2002). Comparison of HER-2/neu status between primary breast cancer and corresponding distant metastatic sites. Ann Oncol.

[B16] Lueftner DI, Dilk H, Henschke P, Geppert R, Dietel M, Stein H, Wernecke K, Possinger K, Heine B (2004). Concordance of HER-2/neu expression of primary breast carcinomas and their metachronous distant metastases: results of a 10 year retrospective search in two university institutes of pathology [abstract 3045]. Breast Cancer Res Treat.

[B17] Regitnig P, Schippinger W, Lindhauer M, Samonigg H, Lax SF (2004). Change of HER-2/neu status in a subset of distant metastases from breast carcinomas. J Pathol.

[B18] Carney WP, Neumann R, Lipton A, Leitzel K, Ali S, Price C (2003). Potential clinical utility of serum HER-2/neu oncoprotein concentrations in patients with breast cancer. Clin Chem.

[B19] Molina R, Jo J, Filella X, Zanon G, Pahisa J, Munoz M, Farrus B, Latre ML, Gimenez N, Hage M (1996). C-erbB-2 oncoprotein in the sera and tissue of patients with breast cancer. Utility in prognosis. Anticancer Res.

[B20] Meng S, Tripathy D, Shete S, Ashfaq R, Haley B, Perkins S, Beitsch P, Khan A, Euhus D, Osborne C (2004). HER-2 gene amplification can be acquired as breast cancer progresses. Proc Natl Acad Sci USA.

[B21] Müller V, Witzel I, Lück HJ, Köhler G, von Minckwitz G, Möbus V, Sattler D, Wilczak W, Löning T, Jänicke F (2004). Prognostic and predictive impact of the HER-2/neu extracellular domain (ECD) in the serum of patients treated with chemotherapy for metastatic breast cancer. Breast Cancer Res Treat.

[B22] Lankiewicz S, Gutierrez B, Böcher O (2006). Quantitative real-time RT-PCR of disseminated tumor cells in combination with immunomagnetic cell enrichment. Mol Biotechnol.

[B23] Walch A, Specht K, Bink K, Zitzelsberger H, Braselmann H, Bauer M, Aubele M, Stein H, Siewert JR, Höfler H (2001). Her-2/neu gene amplification, elevated mRNA expression, and protein overexpression in the metaplasia-dysplasia-adenocarcinoma sequence of Barrett's esophagus. Lab Invest.

[B24] Lüftner D, Lüke C, Possinger K (2003). Serum HER-2/neu in the management of breast cancer patients. Clin Biochem.

[B25] Kong SY, Nam BH, Lee KS, Kwon Y, Lee SE, Seong MW, Lee DH, Ro J (2006). Predicting tissue HER2 status using serum HER2 levels in patients with metastatic breast cancer. Clin Chem.

[B26] Fehm T, Gebauer G, Jeger W (2002). Clinical utility of serial serum c-erbB-2 determinations in the follow-up of breast cancer patients. Breast Cancer Res Treat.

[B27] Ali SM, Leitzel K, Chinchilli VM, Engle L, Demers L, Harvey HA, Carney W, Allard JW, Lipton A (2002). Relationship of serum HER-2/neu and serum CA 15-3 in patients with metastatic breast cancer. Clin Chem.

[B28] Hayes DF, Yamauchi H, Broadwater G, Cirrincione CT, Rodgique SP, Berry DA, Younger J, Panasci LL, Millard F, Duggan DB (2001). Circulating HER-2/erbB-2/c-neu (HER-2) extracellular domain as a prognostic factor in patients with metastatic breast cancer. Clin Cancer Res.

[B29] Esteva FJ, Valero V, Booser D, Guerra LT, Murray JL, Pusztai L, Cristofanilli M, Arun B, Esmaeli B, Fritsche HA (2002). Phase II study of weekly docetaxel and trastuzumab for patients with HER-2-overexpressing metastatic breast cancer. J Clin Oncol.

[B30] Köstler WJ, Schwab B, Singer CF, Neumann R, Rucklinger E, Brodowicz T, Tomek S, Miedermayr M, Hejna M, Steger GG (2004). Monitoring of serum Her-2/neu predicts response and progression-free survival to trastuzumab-based treatment in patients with metastatic breast cancer. Clin Cancer Res.

[B31] Esteva FJ, Cheli CD, Fritsche H, Fornier M, Slamon D, Thiel R, Luftner D, Ghani F (2005). Clinical utility of serum HER-2/neu in monitoring and prediction of progression-free survival in metastatic breast cancer patients treated with trastuzumab-based therapies. Breast Cancer Res.

[B32] Ali SM, Esteva FJ, Fornier M, Gligorov J, Harris L, Kostler WJ, Luftner D, Pichon MF, Tse C, Lipton A (2006). Serum HER-2/neu change predicts clinical outcome to trastuzumab-based therapy [abstract 500]. J Clin Oncol.

[B33] Schondorf T, Hoopmann M, Warm M, Neumann R, Thomas A, Gohring UJ, Carsten E, Mallmann P (2002). Serologic concentrations of HER-2/neu in breast cancer patients with visceral metastasis receiving trastuzumab therapy predict the clinical course. Clin Chem.

[B34] Anderson TI, Paus E, Nesland JM, McKenzie SJ, Borresen AL (1995). Detection of c-erbB-2 related protein in sera from breast cancer patients. Acta Oncol.

[B35] Fehm T, Jäger W, Kraemer S, Sohn C, Solomayer-Meyberg G, Solomayer EF, Kurek R, Wallwienerand D, Gebauer G (2004). Changes of serum HER2 status during clinical course of metastatic breast cancer patients. Anticancer Res.

[B36] Butler TP, Gullino PM (1975). Quantitation of cell shedding into efferent blood of mammary adenocarcinoma. Cancer Res.

[B37] Cristofanilli M, Budd GT, Ellis MJ, Stopeck A, Matera J, Miller MC, Reuben JM, Doyle GV, Allard WJ, Terstappen LW (2004). Circulating tumor cells, disease progression, and survival in metastatic breast cancer. N Engl J Med.

[B38] Hayes DF, Cristofanilli M, Budd G, Ellis MJ, Stopeck A, Miller M, Matera J, Allard WJ, Doyle G, Terstappen L (2006). Circulating tumor cells at each follow-up time point during therapy of metastatic breast cancer patients predict progression-free and overall survival. Clin Cancer Res.

[B39] Hayes DF, Walker TM, Singh B, Vitetta ES, Uhr JW, Gross S, Rao C, Doyle GV, Terstappen LW (2002). Monitoring expression of HER-2 on circulating epithelial cells in patients with advanced breast cancer. Int J Oncol.

[B40] Meng S, Tripathy D, Shete S, Ashfaq R, Saboorian H, Haley B, Frenkel E, Euhus D, Leitch M, Osborne C (2006). uPAR and HER-2 gene status in individual breast cancer cells from blood and tissues. Proc Natl Acad Sci USA.

[B41] Baselga J (2002). Is circulating HER2 more than just a tumor marker?. Clin Cancer Res.

[B42] Molina M, Codony-Servat J, Albanell J, Rojo F, Arribas J, Baselga J (2001). Trastuzumab (Herceptin), a humanized anti-HER2 receptor monoclonal antibody, inhibits basal and activated HER2 ectodomain cleavage in breast cancer cells. Cancer Res.

